# Climate change, tree demography, and thermophilization in western US forests

**DOI:** 10.1073/pnas.2301754120

**Published:** 2023-04-24

**Authors:** Kyle C. Rosenblad, Kathryn C. Baer, David D. Ackerly

**Affiliations:** ^a^Department of Integrative Biology, University of California, Berkeley, CA 94720; ^b^Anchorage Forestry Sciences Laboratory, United States Department of Agriculture, Forest Service Pacific Northwest Research Station, Anchorage, AK 99501; ^c^Department of Environmental Science, Policy, and Management, University of California, Berkeley, CA 94720

**Keywords:** climate change, forests, tree mortality, demography, thermophilization

## Abstract

Climate change is driving widespread changes in ecological communities. Warming temperatures often shift community composition toward more heat-tolerant taxa. The factors influencing the rate of this “thermophilization” process remain unclear. Using 10-y census data from an extensive forest plot network, we show that mature tree communities of the western United States have undergone thermophilization. The mean magnitude of climate warming over the 10-y study interval was 0.32 °C, whereas the mean magnitude of thermophilization was 0.039 °C. Differential tree mortality was the strongest demographic driver of thermophilization, rather than growth or recruitment. Thermophilization rates are associated with recent changes in temperature and hydrologic variables, as well as topography and disturbance, with insect damage showing the strongest standardized effect on thermophilization rates. On average, thermophilization occurred more rapidly on cool, north-facing hillslopes. Our results demonstrate that warming temperatures are outpacing the composition of western US forest tree communities, and that climate change may erode biodiversity patterns structured by topographic variation.

## Significance

Under climate change, ecological communities are becoming dominated by species with higher temperature optima. The rate of this “thermophilization” process is difficult to predict. We show that recent changes in temperature and hydrology have driven thermophilization in western US forests, but that thermophilization rates are lagging behind climate change by roughly tenfold. These trends suggest that forest trees are becoming increasingly mismatched with their environments, potentially threatening ecosystem service provision. We also show that thermophilization is caused by high mortality among species with lower temperature optima, and that thermophilization is occurring more rapidly on north-facing hillslopes and in forests damaged by insects. Our results clarify mechanisms of climate-driven shifts in ecological communities, and these insights can inform forest management.

Global climate change is reorganizing ecological communities (1, 2), often in ways that are difficult to anticipate. For example, drought-driven tree mortality rates are increasing, but it remains challenging to predict when, where, and to which species mortality events will occur (3). Successful natural resource management will depend on improved understanding of the factors governing variation in community responses to climate change.

Although community responses to climate change vary, there are likely underlying commonalities in relation to species’ functional attributes or climatic niches (4). Warming temperatures often increase the relative abundance of heat-tolerant (“thermophilic”) taxa (5). This “thermophilization” process has been documented across many taxa, regions, and spatial scales (6–8). However, thermophilization rates vary widely, and although they are often associated with warming rates, much variation remains unexplained.

Additional factors beyond warming rates could influence thermophilization rates. For example, many organisms, such as plants, exhibit physiological links between their water and temperature regulation mechanisms. Consequently, thermophilization rates may be associated with changes in hydrologic variables (6). Additionally, in forests, canopy disturbance increases penetration of solar radiation into forest understories, which can accelerate changes in climate and community composition (9, 10). Tree species may also be differentially susceptible to biotic factors, such as insect damage, which could influence rates of thermophilization.

Topographic features such as hillslope orientation (i.e., slope and aspect) might also modify thermophilization rates. A site’s hillslope orientation affects the amount of heat received from the sun, with the warmest climates occurring on equator-facing hillslopes and the coolest climates on pole-facing hillslopes. In turn, plant community composition is shaped strongly by hillslope-mediated microclimatic variation (11), with warmer-associated species occurring on more equator-facing hillslopes. It is less clear how hillslope orientation might interact with climate change to affect rates of change in community composition over time.

Another key step toward improved understanding of thermophilization is to examine the demographic processes underlying shifts in community composition. The average climatic niche (or “community temperature index”) of a community can be quantified as the mean of all cooccurring species’ temperature optima, often weighted by a metric of species’ abundance. When metrics that account for each organism’s size (e.g., basal area) are used, a community’s temperature index can be changed by losses (mortality), gains (recruitment), or changes in size (growth) (12, 13). Algebraic decomposition can quantify the contributions of each of these processes to overall thermophilization (12, 13). Quantifying these contributions will be instrumental in predicting the long-term trajectories of ecological communities responding to climate change. For example, recruitment-driven thermophilization indicates that warm-associated propagules are recruiting successfully, which could help stabilize community biomass as climate change progresses. In contrast, mortality-driven thermophilization indicates that cool-associated taxa are being lost and not necessarily replaced by other taxa.

Here, we analyze 10-y changes in tree community composition across 44,992 forest subplots in the western United States from the United States Department of Agriculture Forest Service Forest Inventory and Analysis (FIA) dataset. Using hierarchical Bayesian models that account for latent spatial processes (14), we model the mean temperature indices of tree communities over time as a function of long-term average climate, recent climate change, topography, multiple forms of disturbance, and other predictors. We use these models to address three questions: 1) Are western US forests undergoing thermophilization? 2) What factors modify thermophilization rates? 3) What are the separate contributions of mortality, growth, and recruitment to thermophilization, and how do these contributions respond to potential thermophilization rate modifiers?

## Results

**Fig. 1.**
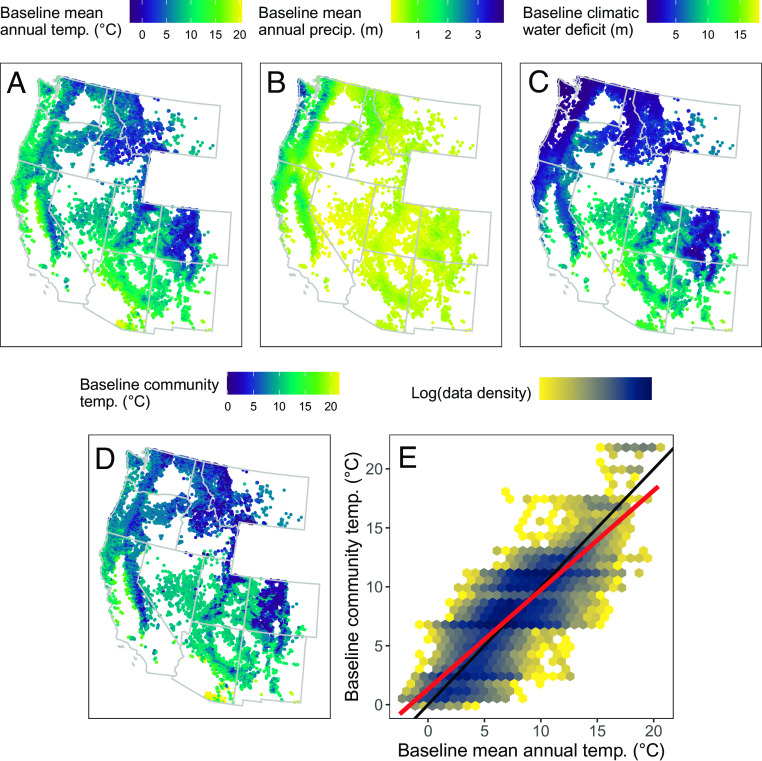
(*A*) Baseline mean annual temperature at 30-arcsecond (roughly 1 km) spatial resolution. (*B*) Baseline mean annual precipitation at 30-arcsecond resolution. (*C*) Baseline climatic water deficit at 1/24-degree (roughly 4 km) spatial resolution. (*D*) Tree community temperature index. In *A*–*D*, the 4 subplot-scale values for each plot are summarized by a single mean value per plot. (*E*) Tree community temperature index vs. baseline mean annual temperature, with hexagons colored by data density. The red trendline, added for illustrative purposes, is generated by simple linear regression (slope = 0.843). The black “one-to-one” line has slope 1 and y-intercept 0.

**Fig. 2.**
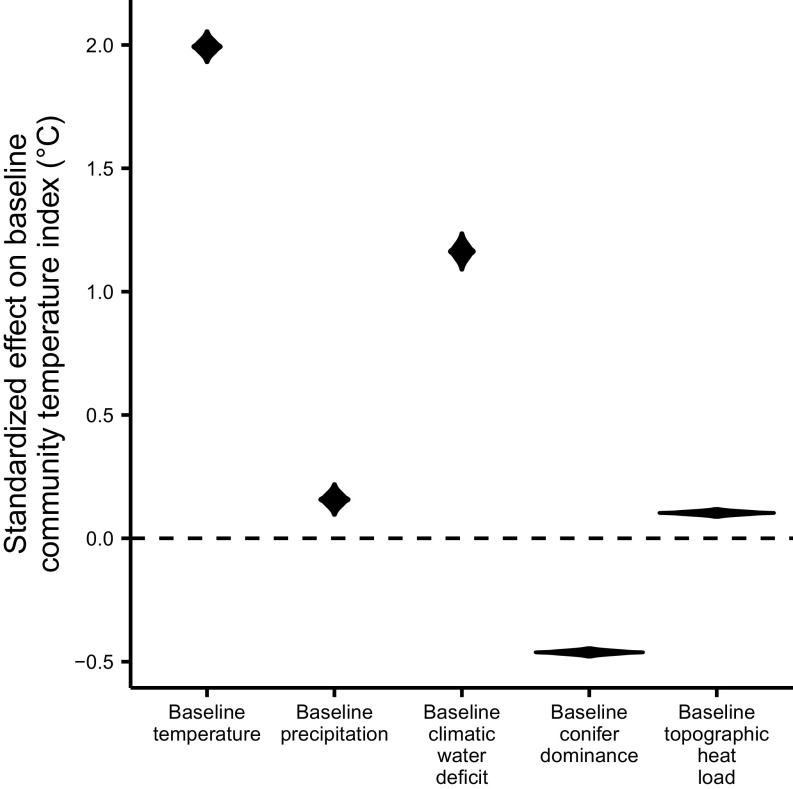
Violin plot of 95% credible intervals for standardized effects of fixed predictors on baseline community temperature index in western US tree communities. Results come from hierarchical Bayesian regression models of tree community temperature index over time. Violins can be interpreted as smoothed, horizontally symmetrical histograms, with the vertical axis representing parameter values and the horizontal axis representing probability density. The total area of each violin is set to be equal, so shorter and wider violins correspond to model parameters for which the posterior probability density is more concentrated around the mean.

In western US forest subplots, baseline community temperature index is greater in warmer regions (0.564 °C community temperature index per 1 °C plot mean annual temperature, in a model containing other climate variables), as well as in regions with greater climatic water deficit (CWD) and precipitation (Figs. 1 and 2). Because continuous predictors were standardized in our models (mean = 0, SD = 1), their effect sizes can be interpreted as °C per SD in the given predictor, thus allowing for comparisons of effect sizes among predictors with different units.

**Fig. 3.**
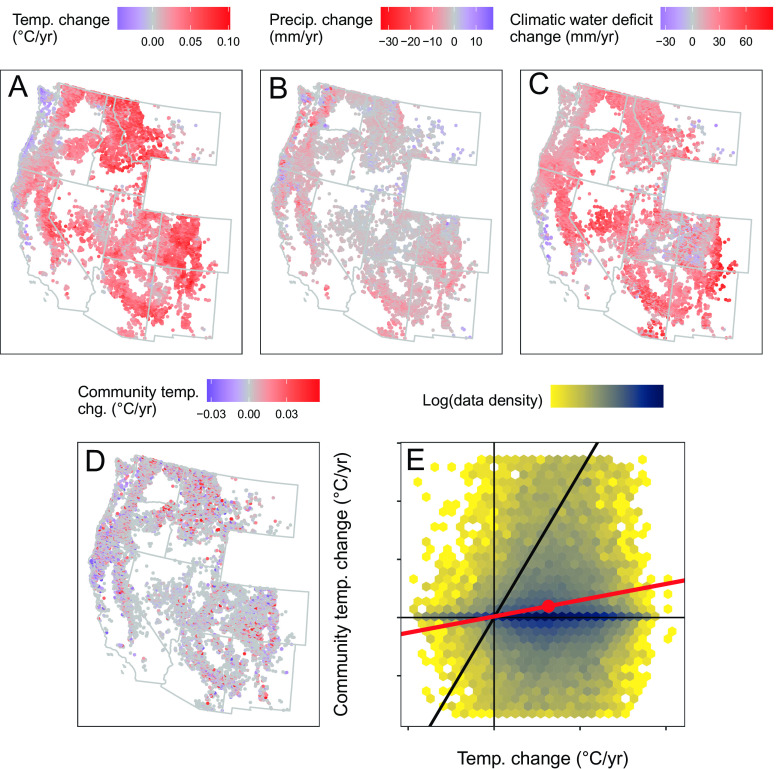
(*A*) Recent 15-y changes in mean annual temperature from gridMET data at 1/24 arcsecond (roughly 4 km) spatial resolution. (*B*) Recent 15-y changes in mean annual precipitation from gridMET. (*C*) Recent 15-y changes in CWD from TerraClimate data at 1/24-degree (roughly 4 km) spatial resolution. (*D*) Recent 10-y changes in tree community temperature index. In *A*–*D*, the 4 subplot-scale values for each plot are summarized by a single mean value per plot. (*E*) Change in tree community temperature index vs. change in mean annual temperature. Each point represents one subplot. The red trendline, added for illustrative purposes, is generated by simple linear regression. The red point represents the mean of the x and y variables. The black “one-to-one” line has slope 1 and y-intercept 0. In *A*–*E*, points below the fifth percentile or above the 95th percentile of change in community temperature index are omitted to improve pattern visibility and color scale perceptibility.

**Fig. 4.**
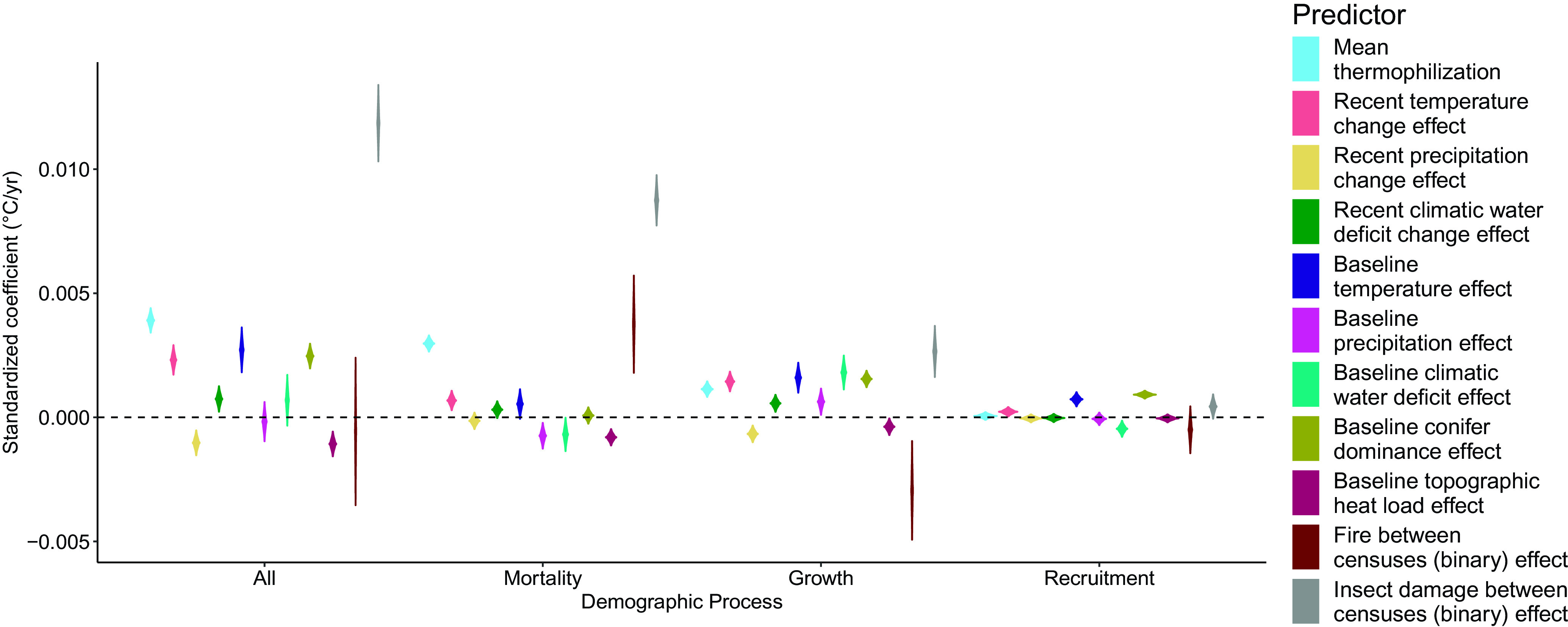
Violin plots of 95% credible intervals for standardized effects of fixed predictors on the magnitude of thermophilization in western US tree communities. Results come from hierarchical Bayesian regression models of tree community temperature index over time. Violins can be interpreted as smoothed, horizontally symmetrical histograms, with the vertical axis representing parameter values and the horizontal axis representing probability density. The total area of each violin is set to be equal, so shorter and wider violins correspond to model parameters for which the posterior probability density is more concentrated around the mean. The interval for mean thermophilization represents the effect size for a binary “T1 vs. T2” predictor that distinguishes between repeat tree censuses. The interval shown for each other predictor represents the effect size for the interaction between the named predictor and the “T1 vs. T2” predictor.

Forested areas in the western United States have warmed on average (Fig. 3; mean temperature change = 0.32 °C over 10 y). Correspondingly, the average subplot-scale tree community has shifted toward warmer-associated taxa—i.e., undergone thermophilization (Figs. 3 and 4). Thermophilization rates are greater in plots that warmed more (0.112 °C thermophilization per 1 °C warming), as well as plots that experienced greater drying, as represented by CWD and precipitation (Figs. 3 and 4). The mean observed thermophilization rate in western US forests is 0.00391 °C/y, whereas the mean observed rate of macroclimatic warming over the same interval is approximately 0.032 °C/y.

The dataset comprised 316,519 trees that survived between censuses (mean = 5.6 per subplot), 64,024 that died (1.1 per subplot), and 35,836 that recruited (0.63 per subplot). Per-tree growth and mortality rates are shown in *SI Appendix*, Fig. S1. Mortality contributed most to thermophilization, followed by growth, with minimal contributions from recruitment (Fig. 4). In this analysis, recruitment is considered as entry into the 12.7 cm diameter-at-breast-height class. In a separate analysis of saplings in the 2.5 cm to 12.7 cm diameter-at-breast-height class, we found that the contribution to thermophilization of recruitment at the 2.5 cm threshold was also negligible (*SI Appendix*, Fig. S2).

Using data from only the portions of each subplot where saplings were recorded (diameter-at-breast-height 2.5 to 12.7 cm), we found that 87% of trees recorded as new 12.7 cm class recruits in the second census had been recorded as saplings in the baseline census. For another 9% of new 12.7 cm class recruits, there was no record of a matching sapling in the baseline census, but there was at least one conspecific seedling with diameter-at-breast-height less than 2.5 cm (which are counted but not marked individually). The remaining 4% of new 12.7 cm class recruits had likely not germinated yet at the baseline census.

Mortality-driven and recruitment-driven thermophilization were greater in plots that warmed more (Fig. 4). Growth-driven thermophilization was greater in plots that increased more in temperature and CWD, as well as plots that decreased more in precipitation (Fig. 4).

Initial community temperature index was greater on warm, south-facing slopes (Fig. 2)—i.e., those with high topographic heat load. Thermophilization was greater on cool, north-facing slopes (Fig. 4). This pattern was driven primarily by mortality (Fig. 4).

Thermophilization was greater at sites where insect damage was observed between surveys (Fig. 4). The effect of this binary predictor on thermophilization rates is stronger than the effect of a one-SD change in any of our continuous predictors, as well as the effect of our binary fire predictor. Similar patterns appear for mortality-driven and growth-driven thermophilization (Fig. 4).

The 95% credible interval for the net effect of fire on thermophilization rates overlaps zero substantially (Fig. 4). However, fire had opposite effects on mortality-driven and growth-driven thermophilization. In subplots that burned between censuses, mortality-driven thermophilization was stronger than that in unburned subplots, whereas growth-driven thermophilization was weaker (Fig. 4).

Thermophilization was greater in conifer-dominated subplots (Fig. 4). This pattern appears in growth-driven thermophilization, as well as in the analysis of recruitment to the 12.7 cm diameter-at-breast-height class (Fig. 4).

All effect sizes are shown in *SI Appendix*, Tables S1–S3, including those not shown in figures. These include the “effects” of fire, insect damage, and recent changes in climate variables on baseline community temperature index. It would be incorrect to interpret these associations as direct causes because the events described by these predictors occurred after baseline community temperature index was measured. Instead, they may reflect causal relationships involving unmeasured covariates (15). Mapped predictions from fixed effects only are shown in *SI Appendix*, Fig. S3, and mapped random effect values are shown in *SI Appendix*, Fig. S4.

## Discussion

Our analyses reveal widespread, fine-grained patterns of change in western US forest tree communities. Subplot-scale community composition has shifted in favor of tree taxa with higher temperature niche means (Figs. 3 and 4). We quantified this thermophilization process using community temperature indices—i.e., weighted averages of species’ climatic niche means in each subplot at each time point. Similarly, baseline community temperature indices are higher in plots with higher multidecadal baseline mean temperatures (Figs. 1 and 2). These results suggest that the observed changes in forest composition were driven at least in part by recent warming.

Thermophilization in western US forests is also associated with drying conditions. This trend is represented in our analyses by the negative effect of precipitation changes and the positive effect of CWD changes (Fig. 4), which were modeled alongside the effect of temperature in our hierarchical Bayesian models. (Variance inflation factors for all predictors were less than 5, although some predictors are moderately correlated, with the greatest Pearson’s correlation of 0.65 occurring between baseline temperature and baseline CWD.) These patterns are consistent with the hypothesis that tree species from warmer climate zones are better adapted to the increased evaporative demands caused by increasing temperatures. This hypothesis is further supported by the positive association we find between baseline community temperature index and baseline CWD in our hierarchical Bayesian regression models.

Although most patterns we found are consistent with warming and drying as drivers of thermophilization, some associations suggest that more complex processes may have operated during or before our study’s timeframe. For example, baseline community temperature index is greater in plots with greater precipitation (Fig. 2). Additionally, although the effect of baseline temperature on baseline community temperature index in our data is near the commonly expected value of 1 in a univariate, ordinary least squares linear regression (Fig. 1*E*), the effect is only 0.564 in our full hierarchical Bayesian regression model, likely reflecting complex causal relationships among community temperature index and the other climatic variables. While effects of precipitation require further consideration, the strongest effects in our data point to warming and drying as the predominant climatic drivers of thermophilization in western US forests.

Thermophilization rates in western US forests are lagging behind macroclimatic warming rates by roughly tenfold. Our results suggest that western US forests are becoming mismatched with their environments. Should the observed trends continue, macroclimatic temperatures will increase by roughly 3 °C on average by the end of the century, whereas the average community temperature index will increase by less than half a degree, thereby adding over 2.5 °C in “climatic debt” (16, 17). This debt will compound any debt these communities may have already accrued due to earlier anthropogenic climate change, migration lags following glacial retreat (18), or other factors that may have restricted species’ realized climatic niches (19). The timeframe of our study coincided with severe drought events in the western United States (20), so it is possible that the patterns we observed are more extreme than future trends will be. However, recently developed climate models indicate that drought is expected to become increasingly frequent and severe in the western United States, and that the timeframe of our study may provide a reasonable preview of upcoming climate change (20). Moreover, to the extent that drought accelerated mortality, it could have led to higher rates of thermophilization, notwithstanding the tenfold lag we observed. Disturbances allow for more rapid forest turnover and in some circumstances may allow plant communities to better track changing environments and minimize climate debt (9).

The consequences of climatic debt may be particularly severe for western US forests because recent thermophilization has been driven primarily by mortality, with little influence from recruitment to the small-tree size class (Fig. 4) or recruitment of smaller saplings (*SI Appendix*, Fig. S2). These patterns indicate that in most subplots, the tree taxa that recruited over the study interval have thermal niches that are no warmer on average than the baseline communities into which they recruited. Instead, thermophilization has mostly resulted from mortality among taxa with the coolest thermal niches. If thermophilization continues to be driven by mortality, then climate change may threaten ecosystem services more strongly than a simple extrapolation of climatic debt might suggest. In this scenario, not only will 3 °C of warming occur, but the 0.5 °C of thermophilization that occurs in response will be due to losses of the most climatically vulnerable species—not recruitment of warmer-associated species that can cope better with warming conditions. These increasingly maladapted forests would likely decline in their ability to provide ecosystem services, such as carbon sequestration (21).

The lack of evidence we find for recruitment-driven thermophilization contrasts with another recent study, which found that hot spots of fecundity and seedling recruitment for western US tree species are shifting toward cooler, moister regions at a rate roughly commensurate with observed climate change (22). This difference in results between studies might be explained by the difference in demographic scope, as our study does not examine seedling dynamics directly, instead examining recruitment of saplings and small trees. If this explanation for the discrepancy in results is correct, then mature tree communities could be primed for more rapid recruitment-driven thermophilization in the coming decades as the current cohorts of seedlings mature. Ackerly et al. (11) predicted that thermophilization driven by seedling recruitment will occur faster on pole-facing hillslopes due to shorter dispersal distances for warmer-associated propagules from communities on adjacent equator-facing hillslopes. Alternatively, our study’s contrasting findings may be explained by the difference in the spatial grain of analysis. There may be a lag between the time when the first seedling of a species recruits anywhere within a new macroclimatic zone and the time when conspecifics have spread to a substantial number of forest plots within that new zone. If this explanation is correct, then the species composition of new recruits in western US forests will likely continue to lag behind climate change.

In addition to climatic predictors, recent thermophilization rates in western US forests are associated with several nonclimatic predictors. Thermophilization rates are roughly three times greater in subplots with recorded evidence of insect damage (Fig. 4). This is the strongest effect size of all thermophilization “rate modifiers” we considered. There is likely at least an indirect causal link between insect damage and thermophilization because some insects, such as bark beetles, attack drought-stressed trees preferentially (23). Insect damage could be an indicator of drought patterns occurring at finer spatiotemporal scales than our predictor variables can capture. Additionally, insect damage may have a direct influence on thermophilization if insect-driven canopy loss accelerates microclimatic change in the understory (24, 25).

We find little evidence for a net effect of fire on thermophilization rates in western US forests over the 10-y study interval (Fig. 4). However, fire appears to have exerted opposite effects on mortality-driven and growth-driven thermophilization. In subplots that burned between censuses, mortality-driven thermophilization was stronger than that in unburned subplots, whereas growth-driven thermophilization was weaker (Fig. 4). A previous study of western US forests found that cool-associated tree taxa have functional traits associated with poor fire tolerance (26), suggesting that fire may disproportionately kill cool-associated taxa. Our results support this prediction. It is unclear why the pattern might be opposite for growth-driven thermophilization. This pattern may reflect features of the postfire environment that disproportionately benefit cool-associated taxa, like decreased competition for moisture, or harm warm-associated taxa, like decreased canopy-mediated thermal buffering during winter (27). It is also possible that the probability of fire occurring during the census interval was influenced by the balance between the growth of cool-associated and warm-associated taxa. Our data on fire occurrence, as well as insect damage, are coarse, and further study is needed regarding the influence of these processes on thermophilization.

Our analyses indicate that topography influences thermophilization in western US forests. Thermophilization was strongest on cool, pole-facing (i.e., north-facing) hillslopes, and this pattern is driven by mortality and growth (Fig. 4). We also find that warmer, more equator-facing sites were occupied by warmer-associated tree taxa at baseline (Fig. 2). For example, our data indicate that at 45°N latitude, the expected difference in community temperature index between a community on a 30° hillslope facing due north and a 30° hillslope facing due south is 0.372 °C. However, it is not clear why thermophilization rates are greatest on pole-facing hillslopes. This pattern would be expected if the rates of change in microclimatic conditions—i.e., exposure to climate change (28)—vary with hillslope orientation (29, 30). Alternatively, thermophilization might be faster on pole-facing hillslopes because tree community sensitivity to climate change [as opposed to exposure (28)] varies with hillslope orientation. Hillslope orientation could have affected historical disturbance regimes (31), which may have shaped variation in resident tree species’ climatic niches (24) and, thus, their sensitivity to climate change.

**Fig. 5.**
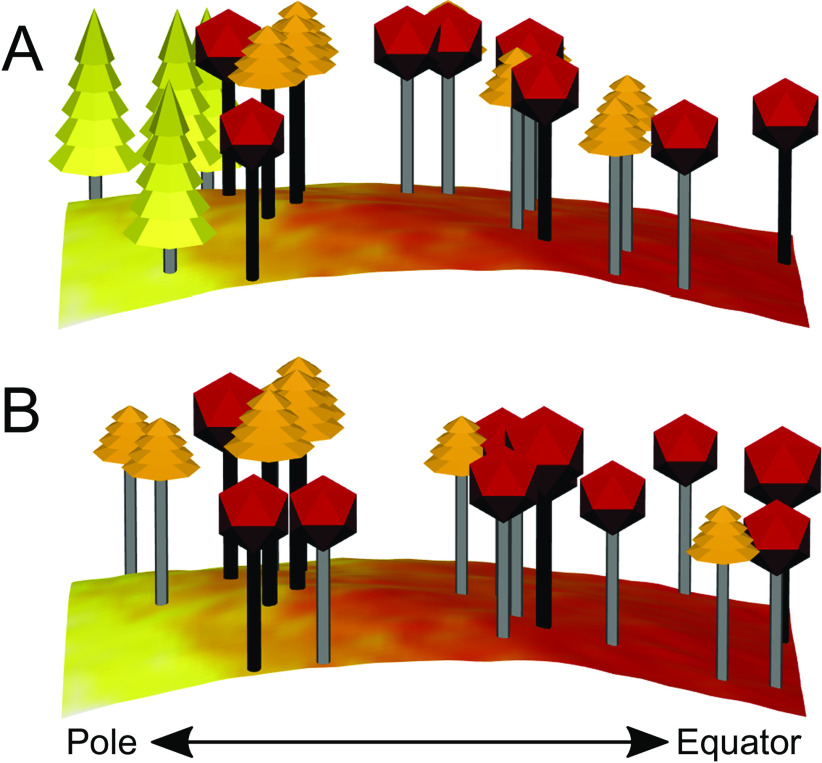
Before (*A*) and after (*B*) views of a topographically heterogeneous landscape occupied by a hypothetical tree community that exemplifies key trends in our data. Effect sizes are magnified for illustrative purposes. Three tree species are shown with three different temperature indices: a low temperature-associated species (five long crown layers shown in yellow, styled after *Abies*), a medium temperature-associated species (four short crown layers shown in orange, styled after *Pinus*), and a high temperature-associated species (an icosahedral crown shown in red, styled after deciduous trees). Trees with black trunks were present at both time points. Trees with gray trunks were only observed in one time point, due either to mortality (present in panel *A* only) or recruitment (present in panel *B* only). Topographic heat load ranges from low (yellow) on the pole-facing hillslope (*Left*) to high (red) on the equator-facing hillslope (*Right*).

Because western US tree communities on pole-facing hillslopes are undergoing thermophilization more rapidly than those of equator-facing neighbors, community temperature indices are becoming more similar between adjacent pole- and equator-facing slopes. In topographically heterogeneous landscapes, this process could drive biotic homogenization—a reduction in spatial turnover of community composition among subplots (11, 32)—as well as declines in landscape-scale species richness. Fig. 5 shows a hypothetical example. This trend could be a bellwether of more pervasive future climate-driven biodiversity loss in topographically heterogeneous landscapes. When biotic homogenization is driven by extirpations, the homogenizing effect of each successive extirpation grows (33). More work is needed to explore the extent and consequences of climate-driven homogenization in topographically heterogeneous forests.

Our study underscores the utility of large datasets with high spatial replication for early detection of biotic responses to climate change, such as thermophilization, accrual of climatic debt, and biotic homogenization. These results could provide a crucial preview of future changes in western US forests that will unfold over longer timescales. We also demonstrate the importance of analyzing the demographic processes underlying thermophilization, showing that western US forests are suffering disproportionately rapid mortality of tree taxa with cool thermal niches, and that new recruits do not have warmer thermal niches than their recipient communities. Additionally, we reveal nonclimatic factors that may modify thermophilization rates, including insect attacks and topographic heat load. Our findings elucidate the mechanisms by which ecological communities respond to climate change, as well as highlight concerning trends in western US forest dynamics that can help inform management strategies.

## Materials and Methods

### Forest Plot Data.

We used the United States Department of Agriculture Forest Service’s FIA Program plot network (34) to quantify changes in western US forest composition over recent 10-y intervals, with baseline plot censuses occurring from 2000 through 2008 and resurveys from 2010 to 2018. FIA uses a randomized systematic sampling approach in which plots are randomly located within hexagonal grid cells of approximately 2,428 ha. We used data collected solely from Phase 2 FIA plots classified as forested (≥10% tree canopy cover), excluding plots that were only partially covered by forest, in the following US states: NM, AZ, CA, NV, UT, CO, ID, OR, WA, and MT. (Wyoming plots had not been recensused at the time of data download.) Each plot comprises four circular subplots, each with a radius of 7.32 m. Within each subplot, the diameter-at-breast-height of each tree with a diameter-at-breast-height of at least 12.7 cm was measured. For all subplots, we disregarded any trees recorded more than 7.32 m from the subplot center so that all sampling units in our analyses were equivalent. In our main analyses, we also disregarded saplings with diameter-at-breast-height less than 12.7 cm, which were recorded in 2.07 m microplots within the main plots. We used the sapling data for supplementary analyses described below. Additional data-filtering protocols are described in *SI Appendix*, *Supplementary Methods*, and all data filtering and preparation steps are shown with comments in our publicly available R code. Forest plots are marked, allowing census protocols to be repeated precisely. Our unit of analysis is the subplot, and our regression models included random effects to account for nonindependence among subplots within the same plot (details below). In total, we analyzed data from 44,992 subplots.

### Quantifying Community Temperature Index.

We quantified temperature index values for each of the 110 species in the dataset using three approaches, which are described in *SI Appendix*, *Supplementary Methods*: modeled niche means, modeled niche optima, and simple niche means. These values are calculated in °C and reflect a measure of the mean or optimal value of mean annual temperature for each species across its geographic range in the western United States. The mgcv R package (35) was used for thermal niche modeling, and CHELSA (Climatologies at high resolution for the Earth's land surface areas) (36, 37) was used for climate data.

To quantify the mean community temperature index in each subplot’s tree community at each timepoint, we calculated the mean temperature index value across all species using each of the three niche modeling methods detailed in *SI Appendix*, *Supplementary Methods*, weighted by the basal area of each species.

### Regression Models of Thermophilization.

Our regression models were built in a Bayesian framework using R-INLA. We used community temperature index in one subplot at one time point (either “T1” or “T2”) as a response variable ( $Y_{\mathrm{ijk}}$ ). The subscript “*i*” denotes the *i*th plot, “*j*” denotes the *j*th subplot, and “*k*” denotes our binary timescale (T1 or T2). We tested for changes over time (i.e., thermophilization) using a binary “T1 vs. T2” indicator variable as one of our predictors ( $x_{1k}$ ). Its coefficient ( $\beta_{1}$ ) represents the mean change in community temperature index between censuses, which are 10 y apart for all sites in our dataset. A zero value of $x_{1k}$ represents T1, and a one value represents T2.

Fixed effects representing climate in our regression models include baseline mean annual temperature, baseline mean annual precipitation, baseline mean annual CWD, and temporal changes in the same three variables. These six plot-level variables are denoted by $x_{2i}$…$x_{7i}$ with coefficients $\beta_{2}$…$\beta_{7}$ , respectively. Topographic heat load for each subplot is denoted by $x_{8ij}$ with coefficient $\beta_{8}$ , and the binary fire and insect damage variables are denoted by $x_{9ij}$…$x_{10ij}$ with coefficients $\beta_{9}$…$\beta_{10}$ , respectively. $x_{11ij}$ , with coefficient $\beta_{11}$ , denotes the subplot’s baseline percent basal area of conifers, which have distinctive temperature and water regulation physiology and represent a large component of western US forests. Each subplot is represented by two observations in the dataset (one at T1 and one at T2), and the values for each of the above predictors are equal in each set of paired T1 and T2 observations. Only the value of $x_{1k}$ (the binary T1 vs. T2 indicator) differs, with the T1 observations receiving a zero value. Consequently, the coefficients $\beta_{2}$…$\beta_{11}$ on their own represent their associations with baseline (T1) community temperature indices. Similarly, the y-intercept ( $\beta_{0}$ ) represents the mean of the T1 observations—i.e., the mean baseline community temperature index.

We also included interactions between $x_{1k}$ (the T1 vs. T2 predictor) and each other predictor to test the modifying effects of these predictors on temporal changes in community temperature index (i.e., thermophilization rates). These interactions are denoted by $x_{1k}x_{2i}\ldots x_{1k}x_{11ij}$ with coefficients $\beta_{12}$…$\beta_{21}$ . For example, in the interaction between $x_{1k}$ (the T1 vs. T2 variable) and $x_{8ij}$ (topographic heat load), the coefficient ( $\beta_{18}$ ) represents the mean effect of topographic heat load on thermophilization rates.

Random effects in our regression models include a subplot-level normally distributed random effect ( $v_{\mathrm{ij}}$ ), as is typically used in linear mixed-effects modeling, and a plot-level spatially covarying random effect $u_{i}$ . The plot-level spatial random effect is modeled using a Matérn covariance structure in a Gaussian Markov Random Field, which is computed as the solution to a stochastic partial differential equation using the finite element method (38).

The full model structure is written below:

$$Y_{ijk}\sim N\left(\mu_{ijk},\sigma^{2}\right),$$

$$\begin{array}{c} \mu_{\mathrm{ijk}}={\beta_{0}+\beta_{1}x}_{1k}+{\beta_{2}x}_{2i}+{\beta_{3}x}_{3i}+{\beta_{4}x}_{4i}+{\beta_{5}x}_{5i}\\ \;+{\beta_{6}x}_{6i}+{\beta_{7}x}_{7i}+{\beta_{8}x}_{8ij}+{\beta_{9}x}_{9ij}+{\beta_{10}x}_{10ij}+{\beta_{11}x}_{11ij}\\ \;+{{\beta_{12}x}_{1k}x_{2i}+\ldots {+\beta_{21}x}_{1k}x_{11ij}+u}_{i}+v_{\mathrm{ij}}, \end{array}$$

$$u_{i}\sim GMRF(0,\Sigma ),$$

$$v_{\mathrm{ij}}\sim N(0,d^{2}).$$

We used R-INLA’s default uninformed (i.e., “flat”) prior probability distributions for all parameters.

Changes in forest composition (including size distributions) over time reflect three distinct demographic processes: mortality, growth, and recruitment of small individuals into the minimum censused size class. In our analyses, “recruitment” denotes entry into the 12.7 cm and above size class, as smaller individuals were not recorded. Consequently, recruitment rates reflect the combined effects of reproduction (both in situ and dispersal from outside the plot), germination and seedling establishment, and seedling and sapling growth to reach this size threshold.

**Fig. 6.**
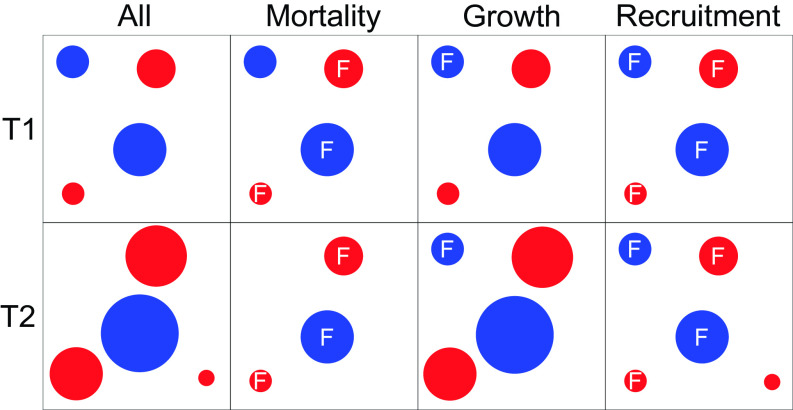
Schematic of our approach to isolating each demographic process’s contribution to changes in tree community temperature index. Circles represent trees of two species (red and blue) as considered by each of the four analytical methods, which are represented by the four columns. The T1 community is considered the same way in all columns, whereas the T2 community differs. Trees marked with “F” are considered to have remained at fixed size between censuses. In the “All” column, all demographic processes (mortality, growth, and recruitment) are considered. In this example, one individual dies, three grow, and one recruits into the community. In the “Mortality” column, the effects of growth and recruitment are excluded, such that any trees present at both time points are held at constant size, and any trees that recruited are ignored. In the “Growth” column, the effects of mortality and recruitment are excluded, such that all trees that died are considered to have survived (but not grown) and all trees that recruited are ignored. In the “Recruitment” column, the effects of mortality and growth are excluded, such that all trees present at the first time point are considered to have remained present at constant size.

To quantify the contributions of each demographic process to thermophilization, we repeated the regression analysis described above with three additional versions of the community temperature index response variable (12, 13), $Y_{\mathrm{ijk}}$ . The predictors and the T1 values of $Y_{\mathrm{ijk}}$ are unchanged in all models, whereas the T2 values of $Y_{\mathrm{ijk}}$ represent the effects of different demographic processes. In the recruitment model, all individuals that recruited between censuses are included at their observed diameter-at-breast-height values, any trees that died are treated as still alive using the diameter-at-breast-height observed in the first census, and any trees that survived are fixed at zero net growth—i.e., their diameter-at-breast-height for the second census is equal to the value observed in the first census. In the “growth” model, all individuals that recruited between censuses are omitted in the second census, all individuals that survived are included with their observed T1 and T2 diameter-at-breast-height values, and mortality is ignored as described above. In thef “mortality” model, new recruits are ignored as described above, net growth for surviving trees is fixed at zero as described above, and any trees that died are recognized as absent in the second census. Fig. 6 shows a visual example of our analytical approach to isolating the effects of the three demographic processes.

We built a separate version of the recruitment model for the sapling data (diameter-at-breast-height 2.5 cm to 12.7 cm) to explore the sensitivity of our results to the 12.7 cm diameter-at-breast-height recruitment threshold. Community-weighted niche means were computed as described above, except only trees in the sapling class were considered. The model structure was identical to that described above.

All continuous predictors were centered and scaled (mean 0, SD 1). Each model was built twice; once with dummy coding for binary predictors (presence or absence of fire and insect damage), and once with weighted effect coding for these predictors. Weighted effect coding of categorical predictors produces, for each category, an estimate of the deviation from the population mean, while accounting for differences in numbers of observations among categories (39). We included this second coding scheme to obtain estimates of population mean thermophilization.

Among the three versions of community temperature index we generated (*SI Appendix*, *Supplementary Methods*), Pearson’s correlation values are all greater than 0.95 (*SI Appendix*, Fig. S5). For simplicity, the main results presented here represent only the “modeled niche mean.”

### Status of New Recruits.

We used the sapling data (diameter-at-breast-height 2.5 cm to 12.7 cm), along with the FIA seedling dataset (diameter-at-breast-height less than 2.5 cm), to check the status of each new recruit to the 12.7 cm diameter-at-breast-height class at the baseline census: present as a sapling, likely present as a seedling (although individual seedlings were not marked), or not yet germinated.

### Data Sources for Predictors.

Our mean annual temperature and mean annual precipitation predictors, which we used to quantify broad, macroclimatic variation among climate zones, were extracted from CHELSA’s (Climatologies at high resolution for the Earth's land surface areas) 1981 to 2010 climatologies (36, 37). We used the temperature change and precipitation change predictors to quantify climate change over a time interval relevant to the observed changes in vegetation. For each plot, we extracted daily temperature and precipitation data at 4 km resolution from gridMET (40) and calculated the difference in mean values between two time intervals, where t is the year of the first census: t−19 through t−5, and t−4 to t+10. These sliding-window measures average over short-term interannual variability to quantify climate change over a 15-y interval, rather than the 10-y interval between tree censuses, and begin earlier to account for potential lagged effects of climate on vegetation (41).

Baseline CWD, as well as recent changes in CWD, was computed from TerraClimate data (42). Annual CWD was computed as the sum of positive monthly CWDs, where deficit equals potential evapotranspiration minus actual evapotranspiration.

In the publicly available FIA dataset, plot geographic coordinates are subject to a “fuzzing and swapping” protocol designed to prevent unauthorized site access and ensure confidentiality of private data (34). “Fuzzing,” which applies to all plots, displaces plot coordinates by up to 0.8 km in a random direction in most cases, or up to 1.6 km for a small subset of plots. “Swapping,” which only affects up to 20% of plots on private land, trades coordinates between plots on private land in the same county. We assume that fuzzing and swapping introduced random noise into our extracted climate data, but not systematic bias.

The remainder of our predictors are generated from within the FIA dataset. Topographic heat load is calculated from slope and aspect, which were measured by field crews for each subplot, as well as latitude, which is provided in fuzzed and swapped forms as described above (43). This variable is a measure of the extent to which energy from the sun warms each microsite. Fire damage and insect damage are binary predictors recorded by field crews that indicate whether each type of damage was observed to have affected at least 25% of the vegetation in each subplot between censuses (or 50% of an individual species’ damage count).

### Software.

We used R software for all data processing, analysis, and visualization, including the following packages: raster (44), R-INLA (45), mgcv (35), ggplot2 (46), patchwork (47), ncdf4 (48), foreach (49), doParallel (50), rgdal (51), spData (52), sf (53), adehabitatHR (54), rgeos (55), usdm (56), and rcartocolor (57). All R scripts and data are available at https://doi.org/10.6078/D1RX4X.

## Supplementary Material

Appendix 01 (PDF)

## Data Availability

Forest inventory, climate, and R code data have been deposited in Rosenblad, Baer, and Ackerly (2023) Code and Data (https://doi.org/10.6078/D1RX4X) (58).
