# Supplementary material for: Climate change, tree demography, and thermophilization in western US forests
Source: Proc Natl Acad Sci U S A. 2023 Apr 24;120(18):e2301754120. doi: 10.1073/pnas.2301754120 (PMC10161004; doi:10.1073/pnas.2301754120)
Supplement: Supplementary file 1 — Appendix 01 (PDF) [file pnas.2301754120.sapp.pdf]

Supplementary Methods

Additional data filtering protocols

In addition to the data filtering described in the main text, we omitted plots with the following attributes from our analyses: plots with evidence of human-assisted tree regeneration, plots where trees were harvested, plots with evidence of anthropogenic disturbance or treatments, plots where all trees present in the first census died, plots where slope and aspect were not recorded, plots that were not fully surveyed at least twice using FIA's nationally standardized protocol, trees that were recorded but fall outside the standardized plot size, and trees that were suspected of being missed, misidentified, or subject to other complicating events in previous surveys.

Thermal niche modeling

The niche modeling input data for each method are species occurrence data from FIA plots within the spatial domain described above. In modeling species' thermal niches, we included trees with some attributes that were excluded from the spatiotemporal community comparisons described above: those less than 12.7 cm DBH, more than 7.32 m from the subplot center, recorded in a plot that did not receive a repeat census, recorded in a plot with missing subplots, and recorded in a plot where the habitat status (forested vs. non-forested) changed over time. The purpose of these data filters was to facilitate standardized spatiotemporal comparisons among plots, which was not necessary for niche modeling.

Two thermal niche modeling methods use a smoothing spline regression approach implemented in the mgcv R package[1]. The response variable is presence or absence of the focal species in each plot, and the predictor is a smooth term for mean annual temperature that uses cubic regression splines. We used a logit link function between predictor and response. For each species, we used the maximum number of knots among smoothing splines supported by the input data. The selection procedure for input data is described below. Smoothing splines were fit by penalized maximum likelihood, where the penalty is a coefficient, lambda (discussed below), multiplied by a "wiggliness" term, the integral over squared second derivatives of the smooth functions. The optimal value of lambda, which determines the balance between under- and over-fitting (or bias and variance), was selected by mgcv's default generalized cross validation (GCV) algorithm.

For each species, regression input data were selected as follows. A minimum convex polygon is circumscribed in geographic space around all plots where the focal species has been recorded as present ("presences"). This polygon is expanded by a buffer that is either 20 km wide or 10% of the maximum distance between any two presences—whichever distance is greater. Then all plots (both presences and absences) within the expanded polygon are selected. (Some species' ranges extend beyond the western US, and we did not include these portions of their ranges.) Mean annual temperature data for each location were extracted from CHELSA's[2-3] 30-arcsecond resolution climatologies averaged over the years 1979-2013. We used the output of this regression model to calculate two quantities: the niche optimum and the niche mean. The niche optimum is the temperature at which the probability of presence is greatest. The niche mean is the mean temperature of occurrence, weighted by the probability of occurrence across the domain of the input data. We estimated this quantity for each species by discretizing the temperature domain in increments of 0.01°C. The key difference between the niche mean and the

niche optimum is that the mean will be weighted by the frequency of occurrence of different climate types, whereas the optimum is less sensitive to this distribution.

We calculated a third measure of temperature index for each species, a “simple niche mean”. This value is the mean temperature of all plots where the focal species has been recorded, weighted by the basal area of the focal species at each of these plots. The basal area values we used are simply the sums of the basal areas of the individual trees present in each subplot. We did not use any of the FIA data set’s estimated variables that pertain to larger spatial areas than the plots themselves.

References

1. Wood, S.N. Generalized Additive Models: An Introduction with R (2nd edition). Chapman and Hall/CRC. (2017).

2. Karger, D. N. et al. Climatologies at high resolution for the earth’s land surface areas. *Sci. Data* 4, 170122 (2017).

3. Karger D.N. et al. Data from: Climatologies at high resolution for the earth’s land surface areas. Dryad Digital Repository. http://dx.doi.org/doi:10.5061/dryad.kd1d4 (2018).

Supplementary Table 1

Predictor	Mean	2.5 Percentile	97.5 Percentile
**Mean thermophilization**	**0.0391**	**0.0341**	**0.0440**
**Intercept**	**7.51**	**7.46**	**7.56**
**Before vs. after (dummy coding)**	**0.0247**	**0.0196**	**0.0299**
**Percent conifer**	**-0.462**	**-0.479**	**-0.445**
**Mean annual temperature (MAT)**	**1.99**	**1.93**	**2.06**
**Mean annual precipitation (MAP)**	**0.157**	**0.0949**	**0.220**
**Mean climatic water deficit (CWD)**	**1.16**	**0.799**	**1.18**
Fire	0.0452	-0.122	0.212
**Insect damage**	**-0.312**	**-0.396**	**-0.228**
**Change in MAT**	**-0.157**	**-0.201**	**-0.113**
**Change in MAP**	**0.0758**	**0.0421**	**0.110**
**Change in CWD**	**0.0947**	**0.0596**	**0.130**
**Topographic heat load**	**0.103**	**0.0867**	**0.119**
**Before vs. after: Percent conifer**	**0.0247**	**0.0197**	**0.0297**
**Before vs. after : MAT**	**0.0272**	**0.0181**	**0.0362**
Before vs. after : MAP	-0.00173	-0.00967	0.00620
Before vs. after : CWD	0.00689	-0.00337	0.0171
Before vs. after : Fire	-0.00569	-0.0355	0.0241
**Before vs. after : Insect damage**	**0.119**	**0.103**	**0.134**
**Before vs. after : Change in MAT**	**0.0231**	**0.0171**	**0.0291**
**Before vs. after : Change in MAP**	**-0.0103**	**-0.0153**	**-0.00523**
**Before vs. after : Change in CWD**	**0.00739**	**0.00223**	**0.0125**
**Before vs. after : THL**	**-0.0107**	**-0.0158**	**-0.00571**

Supplementary Table 1 Legend: Means and 95% credible intervals from estimated posterior distributions of fixed effects in hierarchical Bayesian regression models of tree community weighted temperature index over time. Colons denote interaction terms. All terms except mean thermophilization come from a model that used dummy coding for all binary predictors. Mean thermophilization comes from a model that used weighted effect coding for fire and insect damage. Bold text highlights credible intervals that do not overlap zero.

Supplementary Table 2

Model	Predictor	Mean	2.5 Percentile	97.5 Percentile
**Growth**	**Mean thermophilization**	**0.0114**	**0.00801**	**0.0148**
**Growth**	**Intercept**	**7.51**	**7.45**	**7.57**
**Growth**	**Before vs. after (dummy coding)**	**0.00892**	**0.00537**	**0.0125**
**Growth**	**Percent conifer**	**-0.477**	**-0.494**	**-0.460**
**Growth**	**Mean annual temperature (MAT)**	**1.99**	**1.92**	**2.06**
**Growth**	**Mean annual precipitation (MAP)**	**0.169**	**0.100**	**0.238**
**Growth**	**Mean climatic water deficit (CWD)**	**1.17**	**1.09**	**1.25**
Growth	Fire	0.0629	-0.116	0.242
**Growth**	**Insect damage**	**-0.314**	**-0.407**	**-0.224**
**Growth**	**Change in MAT**	**-0.146**	**-0.194**	**-0.0979**
**Growth**	**Change in MAP**	**0.0738**	**0.0373**	**0.110**
**Growth**	**Change in CWD**	**0.0907**	**0.0524**	**0.129**
**Growth**	**Topographic heat load**	**0.0987**	**0.0826**	**0.115**
**Growth**	**Before vs. after: Percent conifer**	**0.0153**	**0.0119**	**0.0188**
**Growth**	**Before vs. after : MAT**	**0.0161**	**0.00985**	**0.0223**
**Growth**	**Before vs. after : MAP**	**0.00646**	**0.000993**	**0.0119**
**Growth**	**Before vs. after : CWD**	**0.0181**	**0.0110**	**0.0251**
**Growth**	**Before vs. after : Fire**	**-0.0298**	**-0.0503**	**-0.00931**
**Growth**	**Before vs. after : Insect damage**	**0.0266**	**0.0159**	**0.0372**
**Growth**	**Before vs. after : Change in MAT**	**0.0145**	**0.0104**	**0.0186**
**Growth**	**Before vs. after : Change in MAP**	**-0.00652**	**-0.0100**	**-0.00305**
**Growth**	**Before vs. after : Change in CWD**	**0.00563**	**0.00208**	**0.00918**
**Growth**	**Before vs. after : THL**	**-0.00393**	**-0.00740**	**-0.000483**
**Mortality**	**Mean thermophilization**	**0.0296**	**0.0265**	**0.032761711**
**Mortality**	**Intercept**	**7.50**	**7.45**	**7.553203386**
**Mortality**	**Before vs. after (dummy coding)**	**0.0181**	**0.0146**	**0.0215**
**Mortality**	**Percent conifer**	**-0.469**	**-0.486**	**-0.451**
**Mortality**	**Mean annual temperature (MAT)**	**1.98**	**1.91**	**2.05**
**Mortality**	**Mean annual precipitation (MAP)**	**0.136**	**0.0698**	**0.202**
**Mortality**	**Mean climatic water deficit (CWD)**	**1.15**	**1.07**	**1.23**
Mortality	Fire	0.0339	-0.154	0.220
**Mortality**	**Insect damage**	**-0.305**	**-0.400**	**-0.210**
**Mortality**	**Change in MAT**	**-0.184**	**-0.231**	**-0.138**
**Mortality**	**Change in MAP**	**0.0820**	**0.0449**	**0.119**
**Mortality**	**Change in CWD**	**0.0998**	**0.0620**	**0.138**
**Mortality**	**Topographic heat load**	**0.0965**	**0.0797**	**0.113**
Mortality	Before vs. after: Percent conifer	0.000785	-0.00258	0.00415
Mortality	Before vs. after : MAT	0.00532	-0.000772	0.0114
**Mortality**	**Before vs. after : MAP**	**-0.00745**	**-0.0128**	**-0.00212**
Mortality	Before vs. after : CWD	-0.00687	-0.0138	1.11E-05
**Mortality**	**Before vs. after : Fire**	**0.0345**	**0.0145**	**0.0544**
**Mortality**	**Before vs. after : Insect damage**	**0.0875**	**0.0771**	**0.0979**

**Mortality**	**Before vs. after : Change in MAT**	**0.00681**	**0.00279**	**0.0108**
Mortality	Before vs. after : Change in MAP	-0.00130	-0.00469	0.00209
Mortality	Before vs. after : Change in CWD	0.00304	-0.000425	0.00650
**Mortality**	**Before vs. after : THL**	**-0.00817**	**-0.0115**	**-0.00480**
Recruitment	Mean thermophilization	0.000987	-0.000739	0.00271
**Recruitment**	**Intercept**	**7.51**	**7.45**	**7.58**
Recruitment	Before vs. after (dummy coding)	0.000465	-0.00144	0.00236
**Recruitment**	**Percent conifer**	**-0.475**	**-0.493**	**-0.457**
**Recruitment**	**Mean annual temperature (MAT)**	**1.99**	**1.92**	**2.07**
**Recruitment**	**Mean annual precipitation (MAP)**	**0.170**	**0.0944**	**0.245**
**Recruitment**	**Mean climatic water deficit (CWD)**	**1.170**	**1.08**	**1.26**
Recruitment	Fire	0.0494	-0.145	0.243
**Recruitment**	**Insect damage**	**-0.311**	**-0.408**	**-0.214**
**Recruitment**	**Change in MAT**	**-0.144**	**-0.196**	**-0.0911**
**Recruitment**	**Change in MAP**	**0.0708**	**0.0312**	**0.110**
**Recruitment**	**Change in CWD**	**0.0855**	**0.0437**	**0.127**
**Recruitment**	**Topographic heat load**	**0.0952**	**0.0783**	**0.112**
**Recruitment**	**Before vs. after: Percent conifer**	**0.00945**	**0.00760**	**0.0113**
**Recruitment**	**Before vs. after : MAT**	**0.00867**	**0.00532**	**0.0120**
Recruitment	Before vs. after : MAP	8.68E-05	-0.00284	0.00301
**Recruitment**	**Before vs. after : CWD**	**-0.00530**	**-0.00908**	**-0.00152**
Recruitment	Before vs. after : Fire	-0.00434	-0.0153	0.00662
Recruitment	Before vs. after : Insect damage	0.00522	-0.000474	0.0109
**Recruitment**	**Before vs. after : Change in MAT**	**0.00340**	**0.00120**	**0.00561**
Recruitment	Before vs. after : Change in MAP	-0.000626	-0.00249	0.00123
Recruitment	Before vs. after : Change in CWD	-0.000500	-0.00240	0.00140
Recruitment	Before vs. after : THL	-0.000282	-0.00213	0.00157

Supplementary Table 2 Legend: Means and 95% credible intervals from estimated posterior distributions of fixed effects in hierarchical Bayesian regression models of tree community weighted temperature index over time, where effects of individual demographic processes are isolated. "Model" distinguishes three alternate models, each of which reflects the effect on community temperature index of only one demographic mechanism: mortality, growth, or recruitment. Colons denote interaction terms. All terms except mean thermophilization come from a model that used dummy coding for all binary predictors. Mean thermophilization comes from a model that used weighted effect coding for fire and insect damage. Bold text highlights credible intervals that do not overlap zero.

Supplementary Table 3

Predictor	Mean	2.5 Percentile	97.5 Percentile
Mean thermophilization	0.00208	-0.00151	0.00566
**Intercept**	**7.35**	**7.28**	**7.41**
Before vs. after (dummy coding)	0.000870	-0.00282	0.00456
**Percent conifer**	**-0.131**	**-0.165**	**-0.0974**
**Mean annual temperature (MAT)**	**2.41**	**2.33**	**2.50**
**Mean annual precipitation (MAP)**	**0.251**	**0.172**	**0.330**
**Mean climatic water deficit (CWD)**	**0.837**	**0.739**	**0.936**
Fire	0.0863	-0.129	0.302
**Insect damage**	**-0.152**	**-0.257**	**-0.0476**
**Change in MAT**	**-0.183**	**-0.239**	**-0.126**
**Change in MAP**	**0.0859**	**0.0442**	**0.128**
**Change in CWD**	**0.131**	**0.0855**	**0.177**
**Topographic heat load**	**0.0754**	**0.0448**	**0.106**
**Before vs. after: Percent conifer**	**0.00355**	**8.66E-05**	**0.00701**
Before vs. after : MAT	-0.000350	-0.00670	0.00600
Before vs. after : MAP	0.00227	-0.00310	0.00764
Before vs. after : CWD	0.00328	-0.00403	0.0106
Before vs. after : Fire	0.00201	-0.0198	0.0238
Before vs. after : Insect damage	0.00940	-0.000766	0.0196
Before vs. after : Change in MAT	-0.00207	-0.00627	0.00213
Before vs. after : Change in MAP	9.70E-05	-0.00332	0.00351
Before vs. after : Change in CWD	-0.000910	-0.00468	0.00286
**Before vs. after : THL**	**-0.00347**	**-0.00689**	**-4.27E-05**

Supplementary Table 3 Legend: Means and 95% credible intervals from estimated posterior distributions of fixed effects in hierarchical Bayesian regression models of sapling community weighted temperature index over time, where the effect of sapling recruitment (as opposed to growth or mortality) is isolated. Saplings are plants with diameter at breast height greater than or equal to 2.5 cm and less than 12.7 cm. Colons denote interaction terms. All terms except mean thermophilization come from a model that used dummy coding for all binary predictors. Mean thermophilization comes from a model that used weighted effect coding for fire and insect damage. Bold text highlights credible intervals that do not overlap zero.

Supplementary Table 4

Predictor	Mean	Standard Deviation
Mean annual temperature (MAT) (°C)	7.27	3.54
Mean annual precipitation (MAP) (mm)	711	557
Climatic water deficit (CWD) (mm)	5470	3240
Change in MAT (°C)	0.474	0.309
Change in MAP (mm)	-40.7	50.5
Change in CWD (mm)	33.0	22.2
Percent conifer	0.905	0.259
Topographic heat load	0.863	0.139

Supplementary Table 4 Legend: Summary statistics for continuous predictors in hierarchical Bayesian models of community temperature index over time.

Supplementary Figure 1

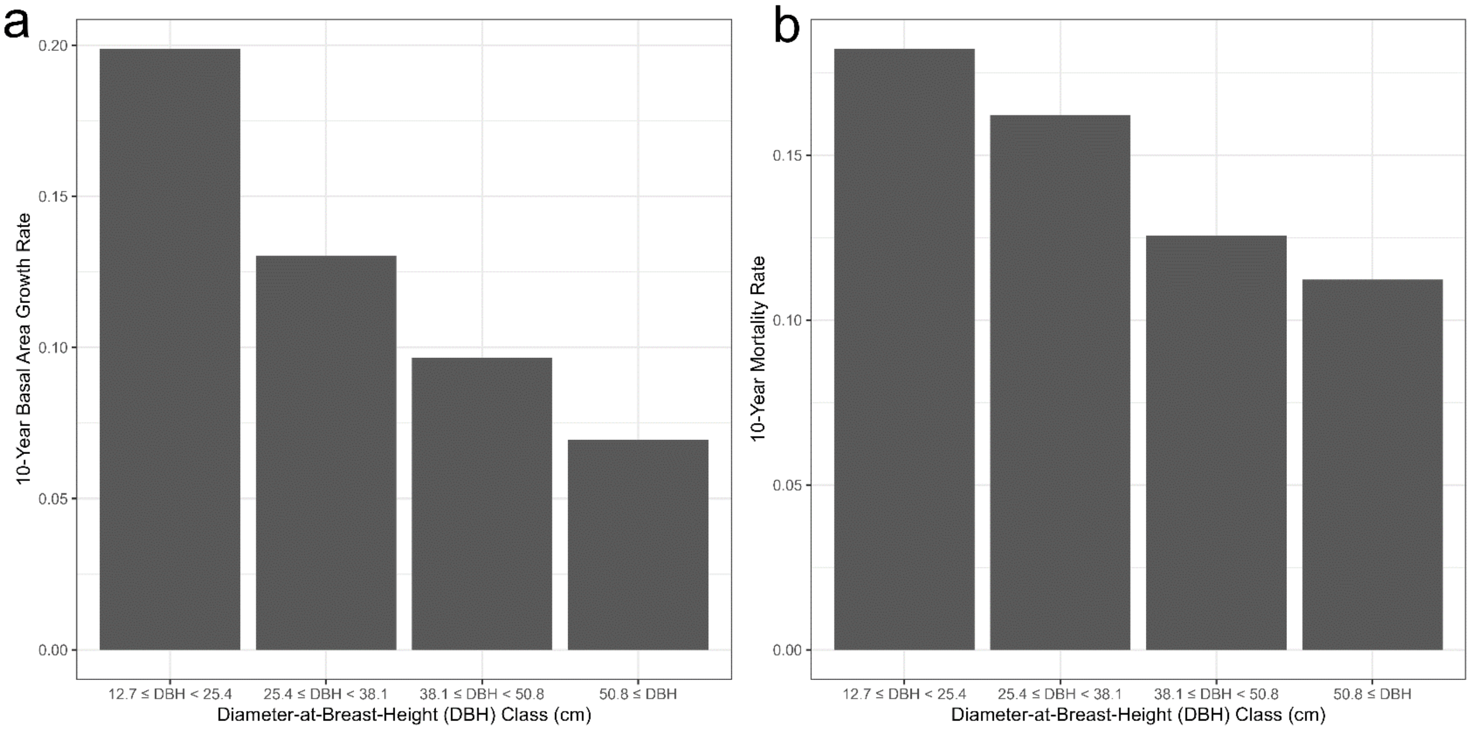

Supplementary Figure 1 Legend: Descriptive demography for the trees in the data set. **a)** Mean 10-year basal area relative growth rates by size class. Growth rate equals the ratio of newly added basal area to initial basal area. **b)** Mortality rates by size class. Mortality rate equals the number of trees that died in each size class divided by the number of live trees in that size class at the baseline census.

Supplementary Figure 2

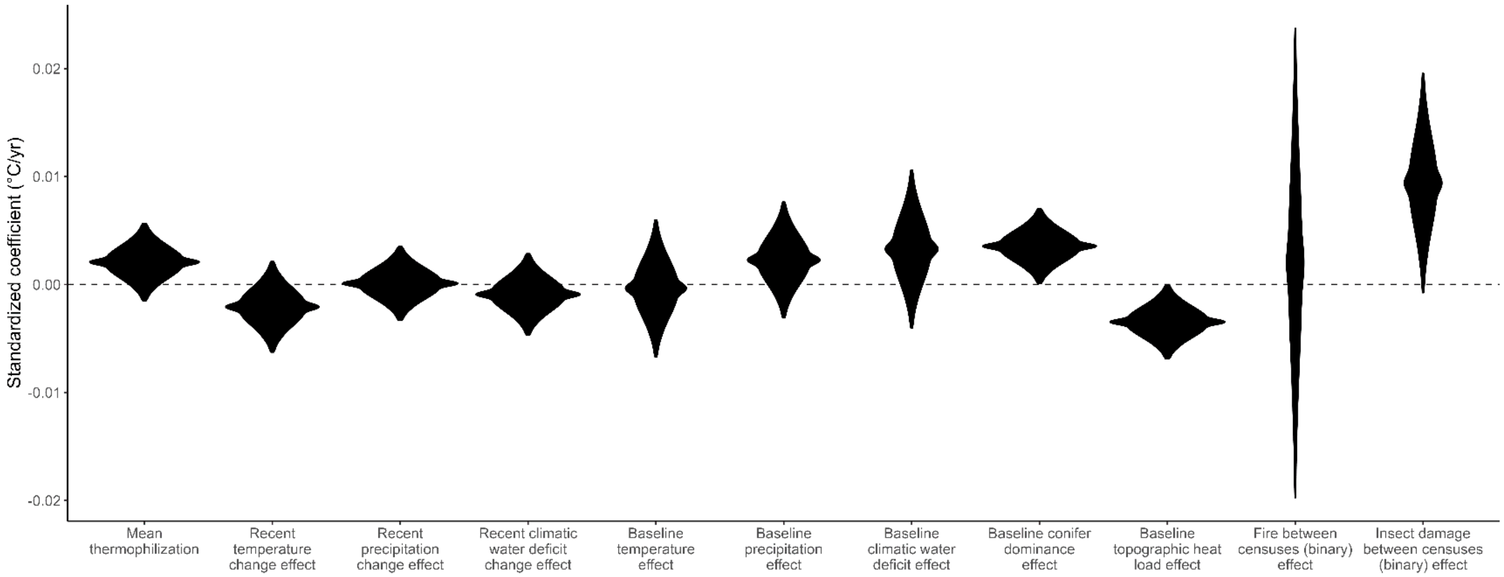

Supplementary Figure 2 Legend: Violin plot of 95% credible intervals for standardized effects of fixed predictors on the magnitude of thermophilization in western US sapling communities. Violins can be interpreted as smoothed, horizontally symmetrical histograms, with the vertical axis representing parameter values and the horizontal axis representing probability density. The total area of each violin is set to be equal, so shorter and wider violins correspond to model parameters for which the posterior probability density is more concentrated around the mean. Saplings are plants with diameter at breast height greater than or equal to 2.5 cm and less than 12.7 cm. Results come from hierarchical Bayesian regression models of sapling community temperature index over time, where the effect of sapling recruitment (as opposed to growth or mortality) is isolated. The interval for mean thermophilization represents the effect size for a binary "T1 vs. T2" predictor that distinguishes between repeat tree censuses. The interval shown for each other predictor represent the effect size for the interaction between the named predictor and the "T1 vs. T2" predictor.

Supplementary Figure 3

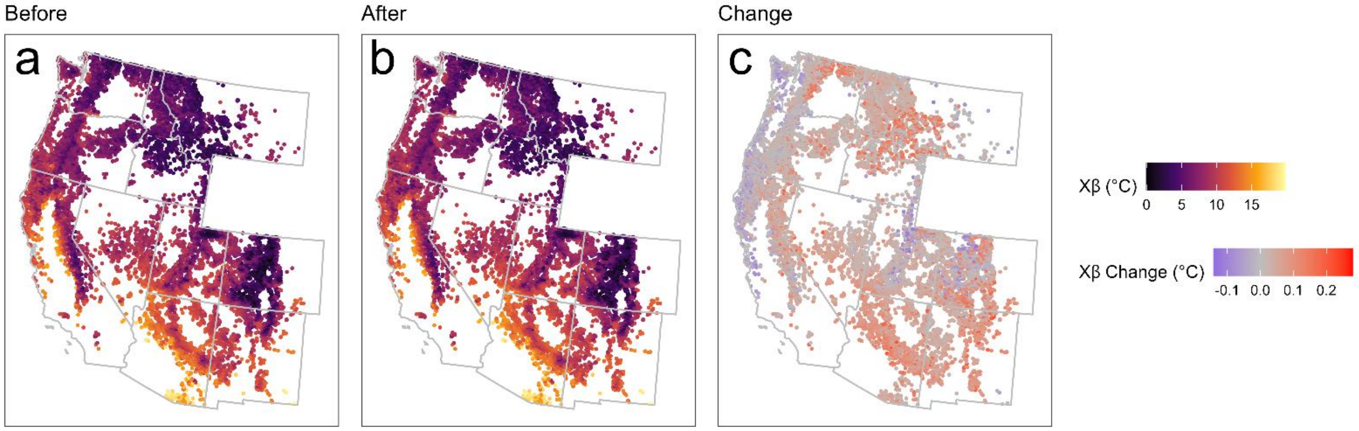

Supplementary Figure 3 Legend: Predicted community temperature index values derived from fixed effects only $(X\beta)$ in the hierarchical Bayesian regression models that consider all demographic processes together. Values shown are for: **a)** the baseline census, **b)** the repeat census, and **c)** the difference between baseline and repeat censuses.

Supplementary Figure 4

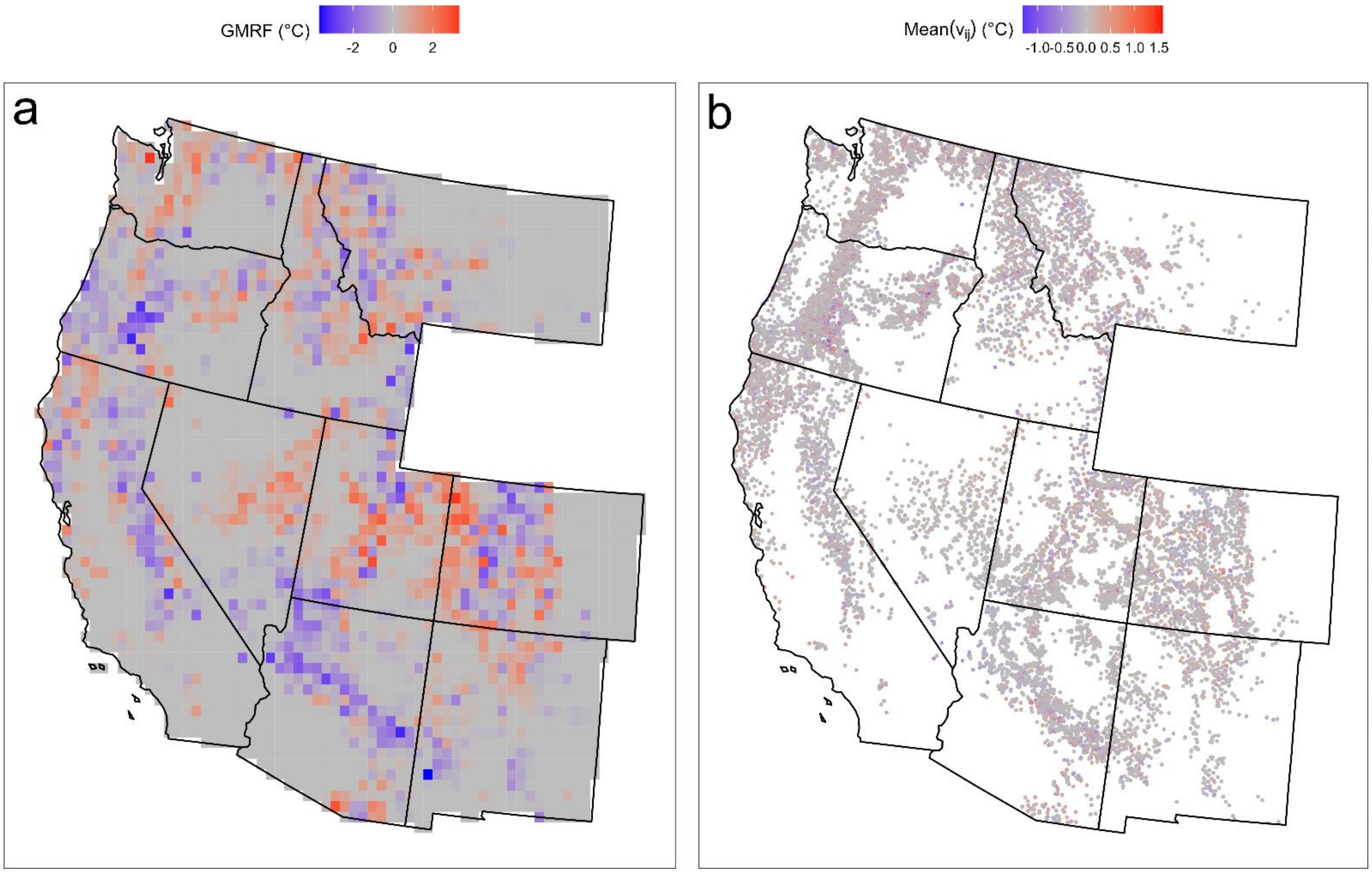

Supplementary Figure 4 Legend: Values of random effects in the hierarchical Bayesian regression models that consider all demographic processes together. **a)** The Gaussian Markov Random Field from which the spatially covarying, plot-level random effect u_i is generated. **b)** Mean values by plot for the subplot-level, normally distributed random effect v_{ij}.

Supplementary Figure 5

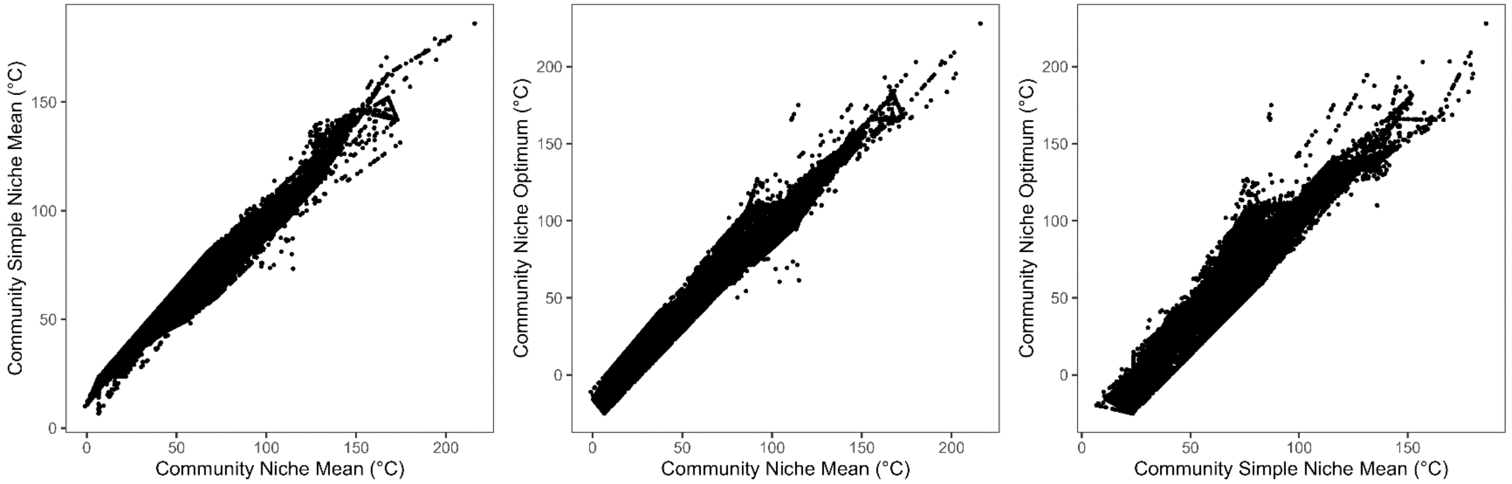

Supplementary Figure 5 Legend: Bivariate relationships among the three versions of community temperature index we generated (see Supplementary Methods).
